# Supplementary material for: Deep convolutional neural networks to predict cardiovascular risk from computed tomography
Source: Nat Commun. 2021 Jan 29;12:715. doi: 10.1038/s41467-021-20966-2 (PMC7846726; doi:10.1038/s41467-021-20966-2)
Supplement: Supplementary file 1 — Supplementary Information [file 41467_2021_20966_MOESM1_ESM.pdf]

Supplementary Materials

Deep convolutional neural networks to predict cardiovascular risk from computed tomography

Roman Zeleznik, Borek Foldyna, Parastou Eslami, Jakob Weiss, Ivanov Alexander, Jana Taron, Chintan Parmar, Raza M. Alvi, Dahlia Banerji, Mio Uno, Yasuka Kikuchi, Julia Karady, Lili Zhang, Jan-Erik Scholtz, Thomas Mayrhofer, Asya Lyass, Taylor F. Mahoney, Joseph M. Massaro, Ramachandran S. Vasan, Pamela S. Douglas, Udo Hoffmann, Michael T. Lu, Hugo J.W.L. Aerts

Supplementary Notes

Supplementary Note 1 Extended failure and outlier analysis

Supplementary Figures

Supplementary Fig. 1 Consort diagrams for training and testing cohorts.
Supplementary Fig. 2 Detailed overview of the Deep Learning System, the available data and the model.
Supplementary Fig. 3 Calcium score Artificial Intelligence vs. Expert Reader.
Supplementary Fig. 4 Calcium score Artificial Intelligence vs. Expert Reader.
Supplementary Fig. 5 Calcium score Artificial Intelligence vs. Expert Reader.
Supplementary Fig. 6 Concordance tables of stratified calcium scores for the testing cohorts.
Supplementary Fig. 7 Examples of automatic segmentations.

Supplementary Tables

Supplementary Table 1 Descriptive statistics for automatically and manually calculated calcium scores.
Supplementary Table 2 AUC values for automatically calculated calcium scores.
Supplementary Table 3 Comparison of automatically and manually calculated calcium scores.
Supplementary Table 4 Accuracy of the first and second deep learning network for localizing and segmenting the heart.
Supplementary Table 5 AUC comparison of event prediction using automatically and manually calculated calcium scores.

Supplementary References

Supplementary Notes

Supplementary Note 1 Extended failure and outlier analysis

To get more insight in the performance of the proposed deep learning system we conducted an in-depth analysis of cases with extreme differences between automatically predicted and manual calcium scores. Therefore we looked into subjects where the predicted calcium score was zero and the human calcium score was above 100 or vice versa, which was the case in 32 out of 5,521 subjects.

In the majority of the cases the deep learning system missed a plaque (n=17), where in one case the missed plaque was close to the valve, one case had severe motion artifacts and in the rest of the cases no obvious reason could be found for the misses. In eight cases the deep learning system segmented a wrong calcium object. In three of these cases noise was segmented in scans of poor image quality, in further three cases valve calcification was segmented, in one case a metal artifact was segmented and in one case a lymph node close to the heart was segmented.

In four cases the human reader missed calcium which where segmented correctly by the deep learning system. In one case the human reader segmented a wrong calcium object while the deep learning system correctly did not segment it. In one case we accidentally included a contrast enhanced scan in our test cohort and the deep learning system segmented a wrong area. Unfortunately, this case was not excluded in our preprocessing image quality assessment. One case had very poor image quality with high noise and motion artifacts which led to human over-segmenting and deep learning under-segmenting coronary calcium.

Manual assessment of segmentation results showed the most common area for segmentation errors was near the aortic and mitral valves. Distinguishing coronary and valvular calcium can be challenging even for humans on ECG-gated CTs.

As shown in Supplementary Fig. 7, the proposed system was able to handle metal artifacts in CT scans in most but not all cases. Current clinical guidelines recommend applying the coronary artery calcium score in patients without known cardiovascular disease[1], and the value of the calcium score in patients with metal in the heart from past cardiac interventions (e.g. prior coronary artery bypass graft, coronary artery stents, or pacemakers) is not clear.

Supplementary Figures

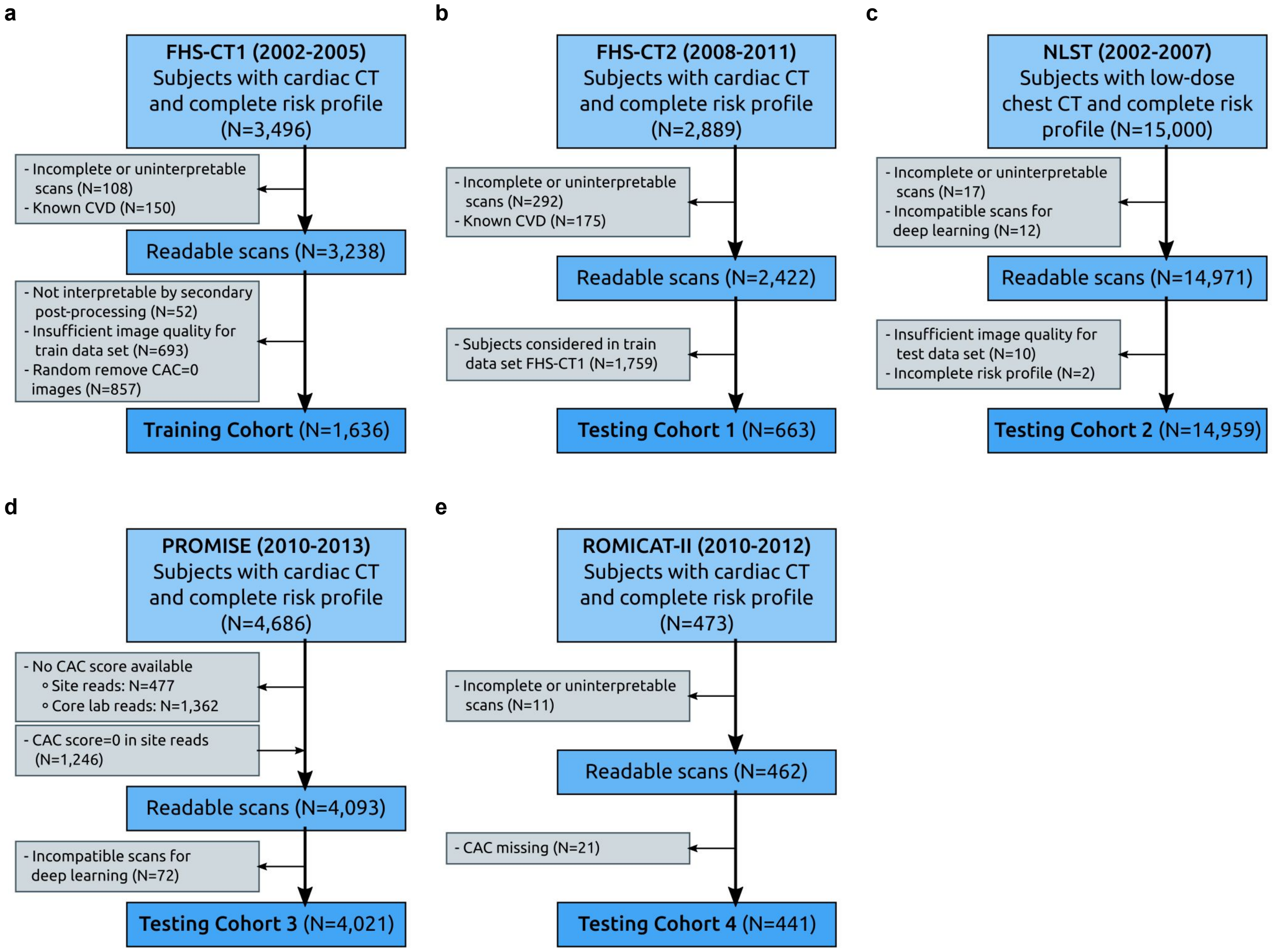

Supplementary Fig. 1 Consort diagrams for training and testing cohorts. **a** FHS-CT1[2]: Subset of the community-based multidetector computed tomographic Heart Study (FHS) with scans from the seventh examination cycle of the Offspring Cohort or first examination cycle of the Third Generation Cohort, taken between 2002 and 2005. 857 scans with no CAC present were randomly selected and removed to balance the number of scans with and without CAC in the training cohort. **b** FHS-CT2[2]: Subset of FHS with participants from the second examination cycle of the Third Generation Cohort, taken between 2008 and 2011, of patients which were not included in FHS-CT1. **c** Subset of the low-dose chest screening CT group of the National Lung Screening Trial (NLST)[3]. **d** Subset of the intervention arm of the Prospective Multicenter Imaging Study for Evaluation of Chest Pain (PROMISE)[4]. **e** Subset of cardiac CT imaging arm of the multicenter Rule Out Myocardial Infarction using Computer-Assisted Tomography II (ROMICAT-II)[5] trial. CT: Computed tomography; CVD: Cardiovascular disease; CAC: Coronary artery calcium.

a

b

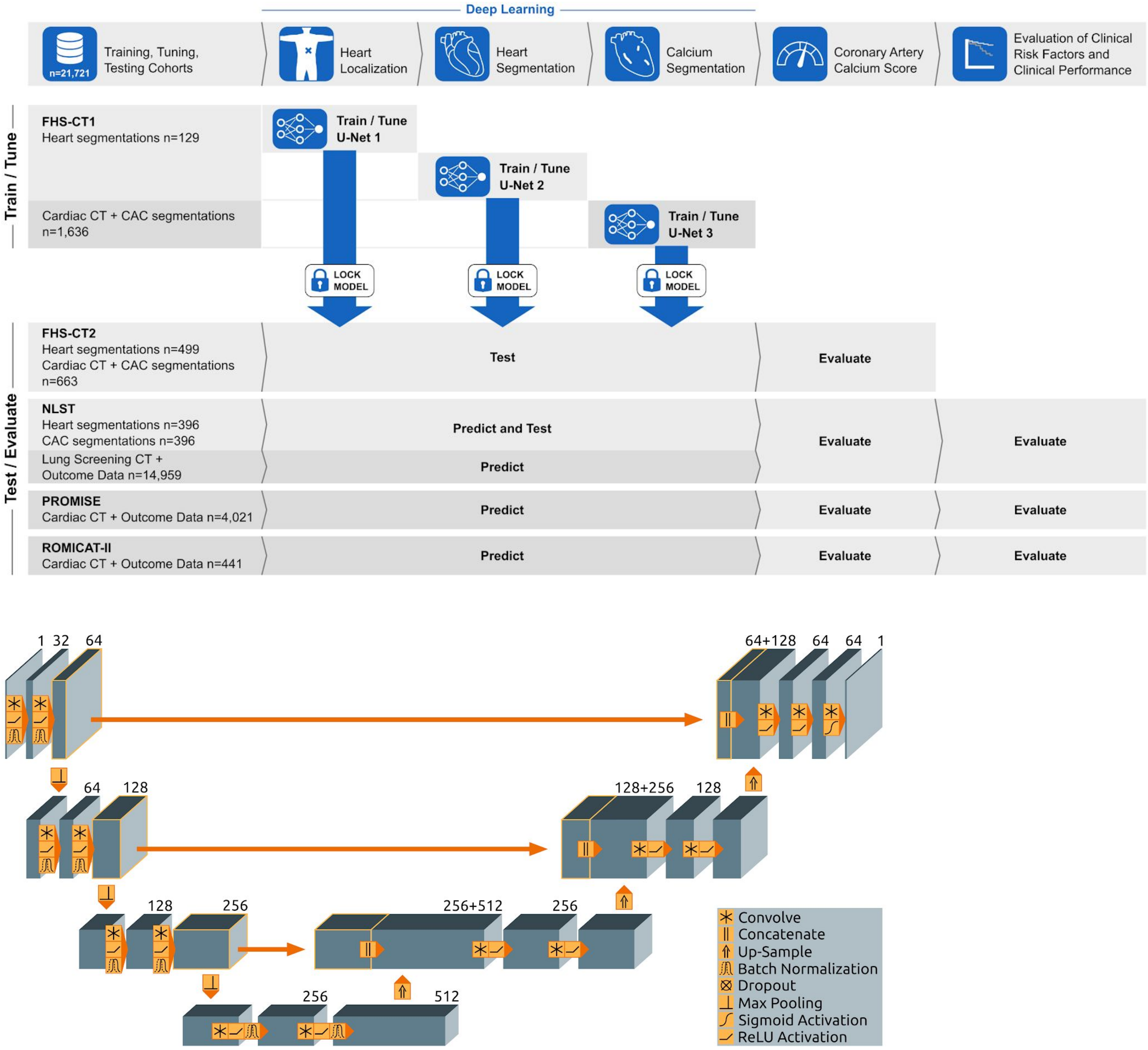

Supplementary Fig. 2 Detailed overview of the Deep Learning System, the available data and the model. **a** Detailed overview of the cohorts and their usage for training, tuning and testing. In total 21,720 CT scans were available to train, tune and test the three deep learning models. The training cohort FHS-CT1 was used to train and tune the three deep learning models. The final models were locked and tested against manual segmentations from expert readers in our independent testing cohorts FHS-CT2 and a subset of NLST. We predicted coronary calcium segmentations in all four testing cohorts and computed the calcium risk score. To evaluate the predictive value for clinical outcomes we used our testing cohorts NLST, PROMISE and ROMICAT-II. **b** Extended U-Net architecture for segmenting coronary calcium. The original proposed U-Net was extended by batch normalization layers in the contracting path (left side) for better generalizability of the model. FHS-CT1[2]: Subset of the community-based multidetector computed tomographic Heart Study (FHS) with scans from the seventh examination cycle of the Offspring Cohort or first examination cycle of the Third Generation Cohort, taken between 2002 and 2005; FHS-CT2[2]: Subset of FHS with participants from the second examination cycle of the Third Generation Cohort, taken between 2008 and 2011; NLST[3]: National Lung Screening Trial; PROMISE[4]: Prospective Multicenter Imaging Study for Evaluation of Chest Pain; ROMICAT-II[5]: Rule Out Myocardial Infarction using Computer-Assisted Tomography II trial; CAC: Coronary artery calcium; CT: Computed tomography.

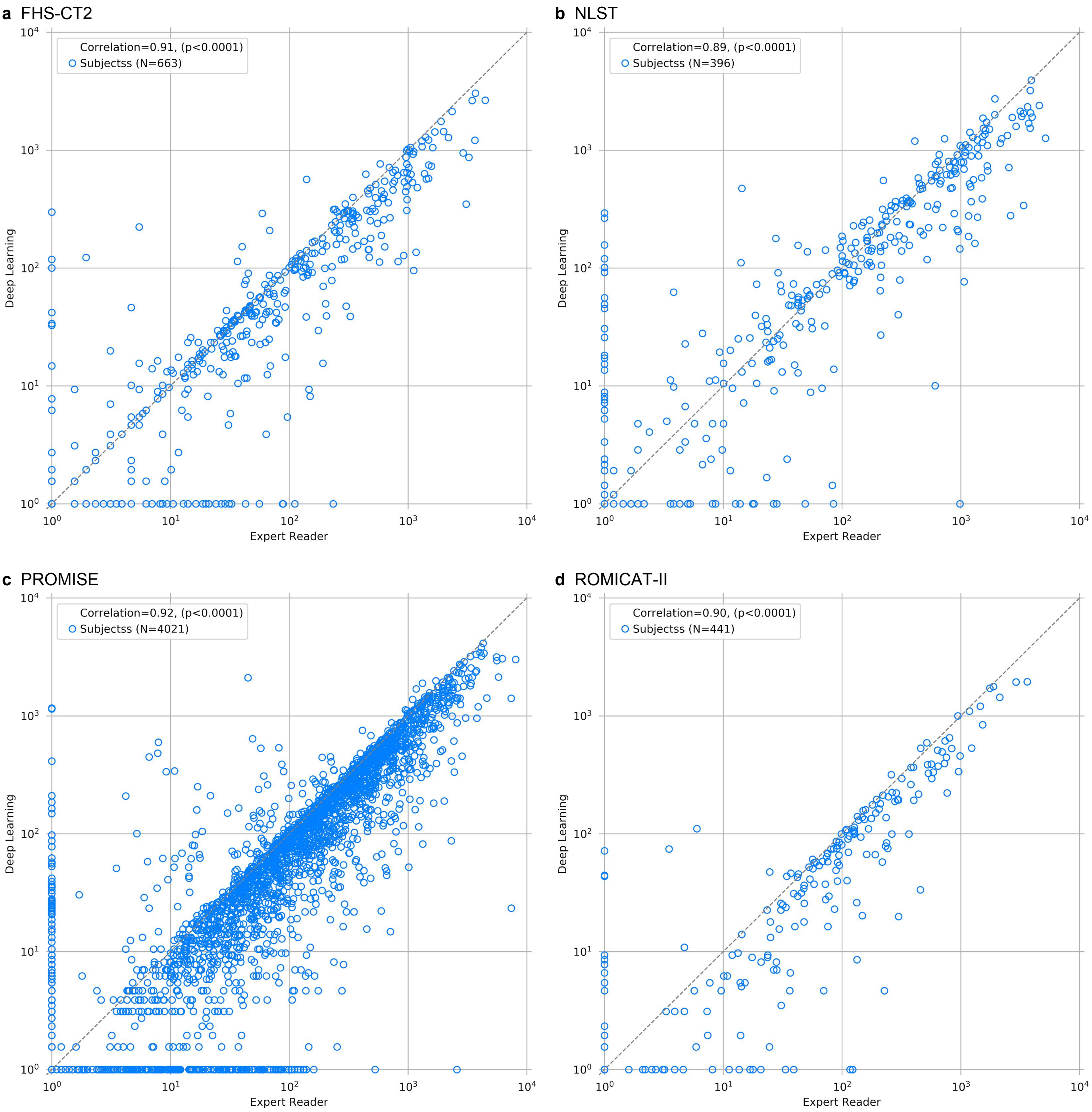

Supplementary Fig. 3 Calcium score Artificial Intelligence vs. Expert Reader. Spearman correlation coefficient with associated two-sided p-value were estimated for the comparison of expert reader and artificial intelligence calculated calcium scores for test cohorts **a** Framingham Heart Study (FHS-CT2)[2], **b** National Lung Screening Trial (NLST)[3], **c** Prospective Multicenter Imaging Study for Evaluation of Chest Pain (PROMISE)[4], and **d** Rule Out Myocardial Infarction using Computer Assisted Tomography (ROMICAT-II)[5]. All p-values were <0.0001.

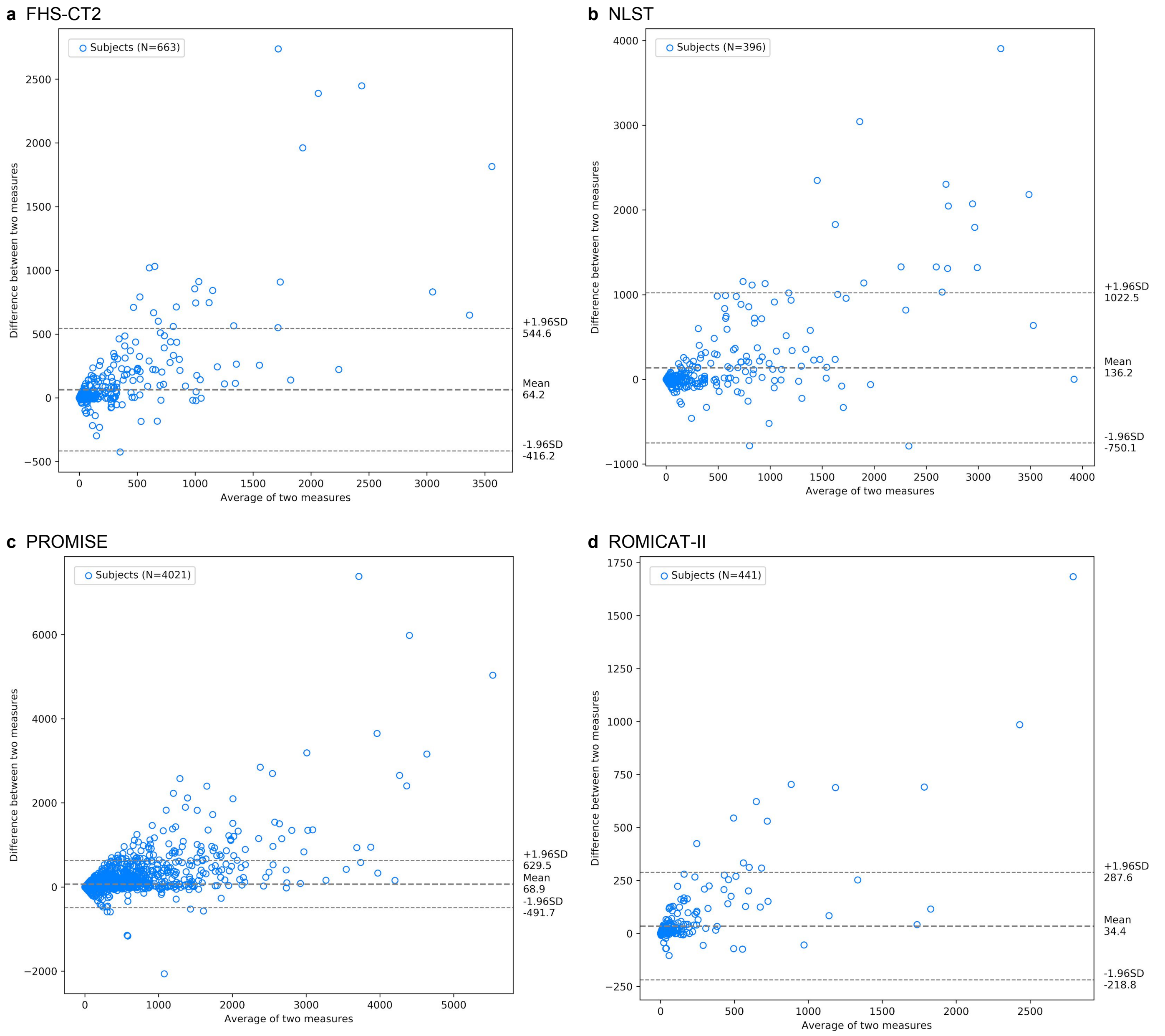

Supplementary Fig. 4 Calcium score Artificial Intelligence vs. Expert Reader. Comparison of expert reader and artificial intelligence calculated calcium scores for test cohorts **a** Framingham Heart Study (FHS-CT2)[2], **b** National Lung Screening Trial (NLST)[3], **c** Prospective Multicenter Imaging Study for Evaluation of Chest Pain (PROMISE)[4], and **d** Rule Out Myocardial Infarction using Computer Assisted Tomography (ROMICAT-II)[5].

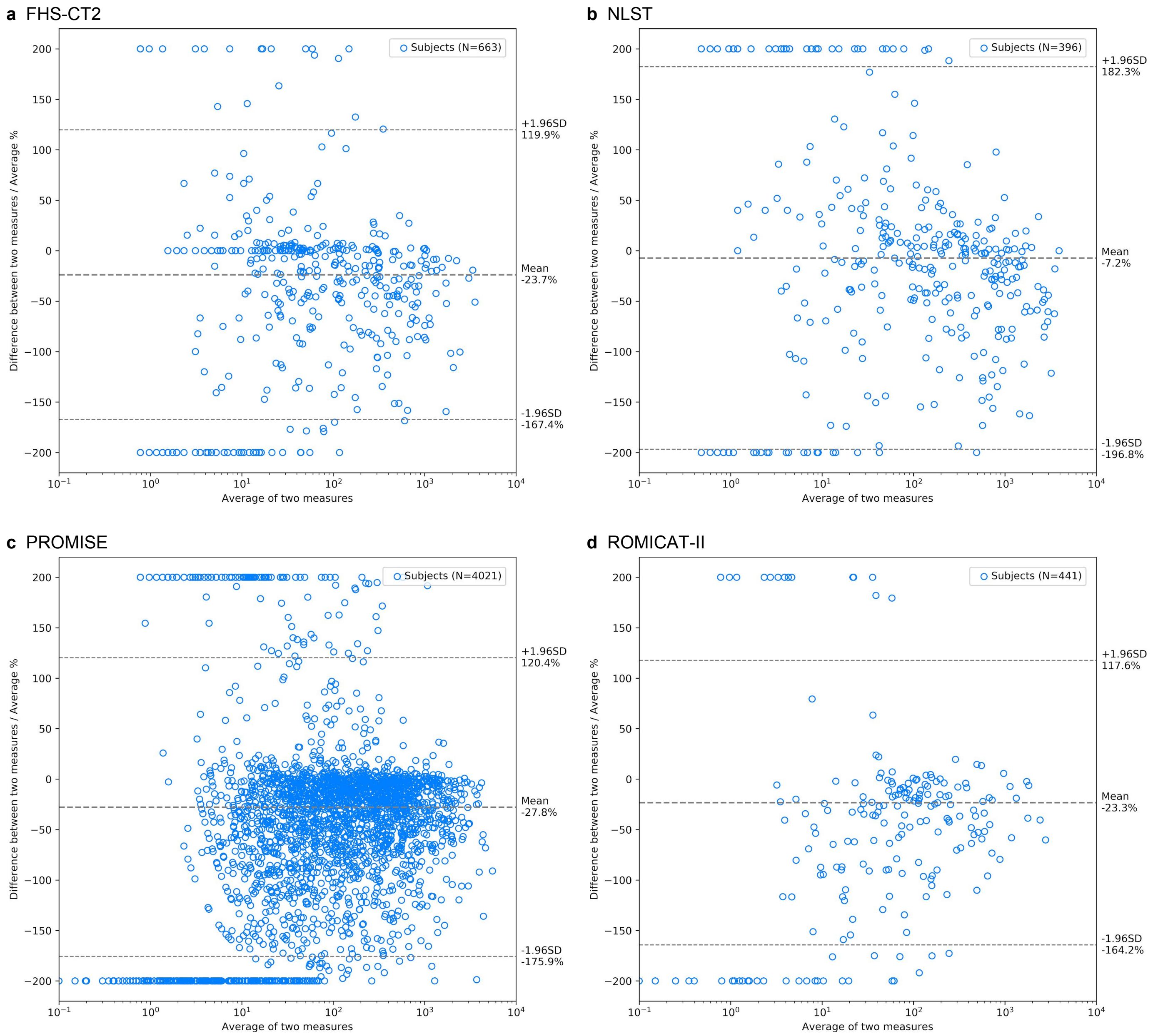

Supplementary Fig. 5 Calcium score Artificial Intelligence vs. Expert Reader. Comparison of expert reader and artificial intelligence calculated calcium scores for test cohorts **a** Framingham Heart Study (FHS-CT2)[2], **b** National Lung Screening Trial (NLST)[3], **c** Prospective Multicenter Imaging Study for Evaluation of Chest Pain (PROMISE)[4], and **d** Rule Out Myocardial Infarction using Computer Assisted Tomography (ROMICAT-II)[5].

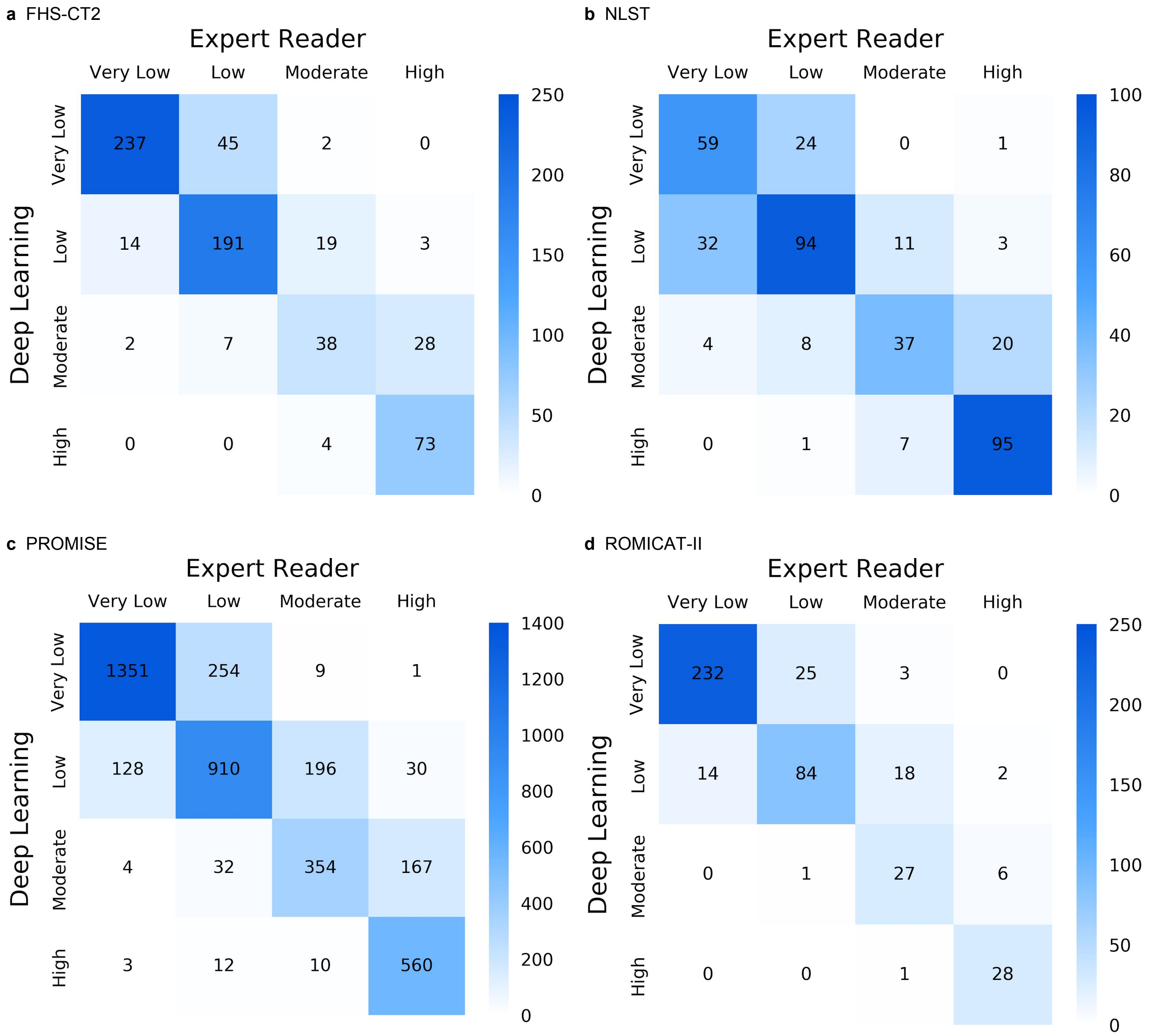

Supplementary Fig. 6 Concordance tables of stratified calcium scores for the testing cohorts. **a** Framingham Heart Study (FHS-CT2)[2]. **b** National Lung Screening Trial (NLST)[3]. **c** Prospective Multicenter Imaging Study for Evaluation of Chest Pain (PROMISE)[4]. **d** Rule Out Myocardial Infarction using Computer Assisted Tomography (ROMICAT-II)[5]. Calcium risk scores are stratified in categories: Very low: 0; Low: 1-100; Moderate: 101-300; **High:** >300[21].

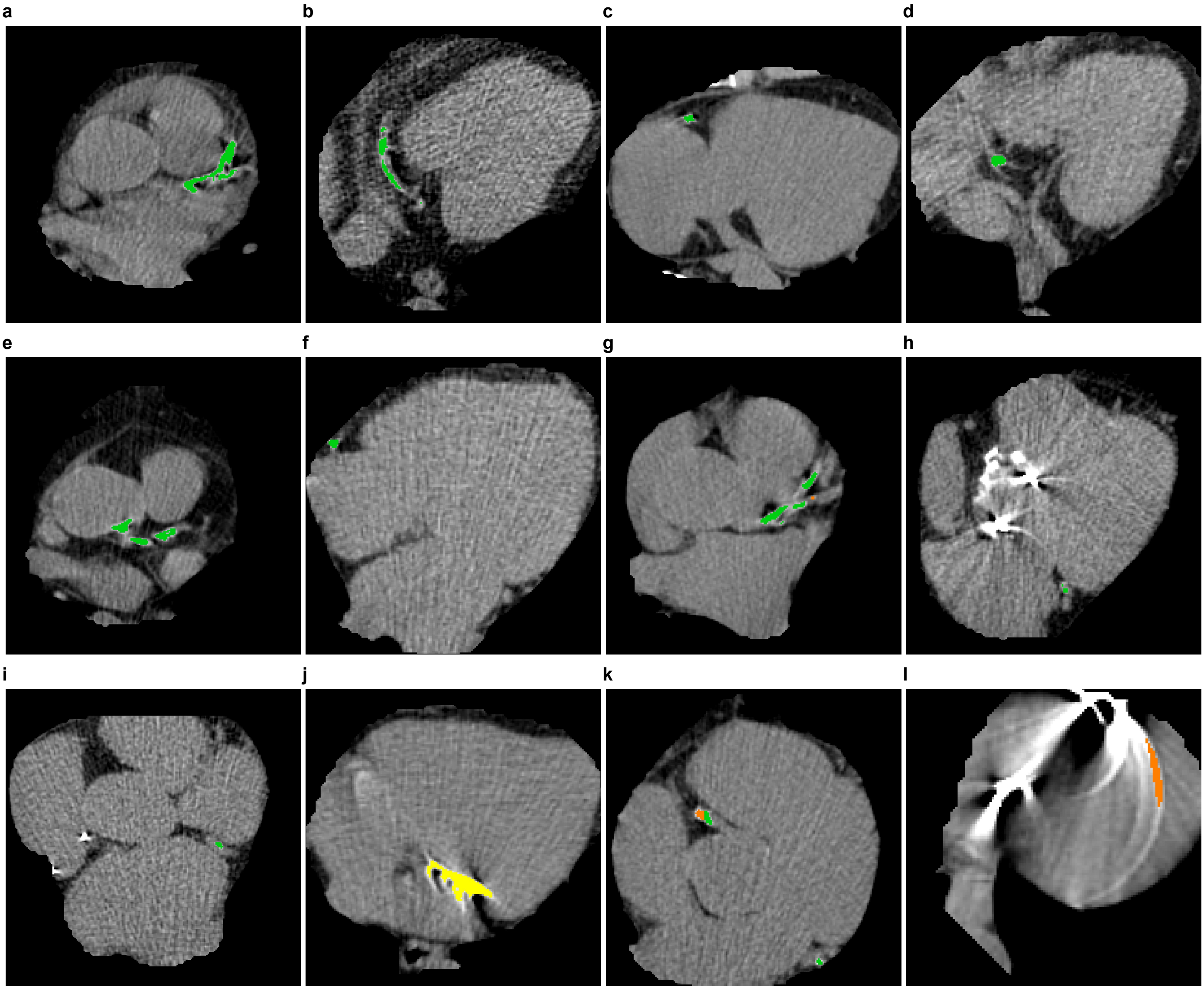

Supplementary Fig. 7 Examples of automatic segmentations. The images show **a - f** correct segmented coronary calcium (green), **g** correct segmented coronary calcium (green) and missing coronary calcium (orange), **h** an artificial valve (white) and correct segmented coronary calcium (green), **I** a pacemaker (white dots) and correct segmented coronary calcium (green), **j** a mitral valve calcification that was mistakenly segmented by a reader (yellow) but was not labeled by the deep learning system, **k** correct segmented coronary calcium (green) and missing coronary calcium (red), **l** falsely segmented part of an artefact (red).

Supplementary Tables

Supplementary Table 1 Descriptive statistics for automatically and manually calculated calcium scores.

**Automatic CAC score***	**FHS-CT2 (n=663)**	**NLST (n=14,959)**	**PROMISE (n=4,021)**	**ROMICAT-II (n=441)**
Continuous	118.7±303.34	359.1±717.4	150.1±353.8	72.1±230.1
0	284 (42.84)	3,613 (24.2)	1,615 (40.2)	260 (59.0)
1-100	227 (34.24)	4,730 (31.6)	1,264 (31.4)	118 (26.8)
101-300	75 (11.31)	2,373 (15.9)	557 (13.9)	34 (7.7)
>300	77 (11.61)	4,243 (28.4)	585 (14.5)	29 (6.6)
**Manual CAC score***	**FHS-CT2 (n=663)**	**NLST (n=396)**	**PROMISE (n=4,021)**	**ROMICAT-II (n=441)**
Continuous	182.94±476.74	429.8±822.4	219.0±537.9	106.4±331.3
0	253 (38.16)	95 (24.0)	1,486 (37.0)	246 (55.8)
1-100	243 (36.65)	127 (32.1)	1,208 (30.0)	110 (24.9)
101-300	63 (9.50)	55 (13.9)	569 (14.2)	49 (11.1)
>300	104 (15.69)	119 (30.1)	758 (18.9)	36 (8.2)

CAC: Coronary Artery Calcium; FHS-CT2[2]: Framingham Heart Study, participants from the second examination cycle of the Third Generation Cohort; NLST[3]: National Lung Screening Trial; PROMISE[4]: Prospective Multicenter Imaging Study for Evaluation of Chest Pain; ROMICAT-II[5]: Rule Out Myocardial Infarction using Computer Assisted Tomography II. *Continuous CAC scores are presented as mean ± standard deviation; Stratified CAC scores are presented as number of subjects with percentage of cohort in parentheses.

Supplementary Table 2 AUC values for event prediction based on automatically calculated calcium scores.

	**AUC**	**SD**	**95%CI**	**P-value**
**NLST:** n=14,959; Events: ASCVD death, n=288	0.6912	0.0149	0.66-0.72	<0.0001
**NLST:** n=14,959; Events: ACM death, n=1,085	0.6309	0.0089	0.61-0.65	<0.0001
**PROMISE:** n=4,021; Events: ACM, MI, UA, n=130	0.6622	0.0237	0.62-0.71	<0.0001
**ROMICAT-II:** n=441; Events: ACS, n=38	0.8308	0.0349	0.76-0.90	<0.0001

NLST[3]: National Lung Screening Trial; PROMISE[4]: Prospective Multicenter Imaging Study for Evaluation of Chest Pain; ROMICAT-II[5]: Rule Out Myocardial Infarction using Computer Assisted Tomography II; AUC: Area under the curve; SD: Standard deviation; CI: Confidence interval; ASCVD: Atherosclerotic cardiovascular disease; ACM: All cause mortality; MI: Myocardial Infarction; UA: Unstable angina; ACS: Acute coronary syndrome. All p-values were calculated using a two-sided Wilcoxon Test.

Supplementary Table 3 Comparison of automatically and manually calculated calcium scores.

Cohort	ICC - 95%CI		Spearman's Correlation - P-value		Kappa
FHS-CT2, n=663	0.800	0.77-0.83	0.912	$<2.2\times10^{-16}$	0.726
NLST, n=396	0.769	0.73-0.81	0.892	$<2.2\times10^{-16}$	0.618
PROMISE, n=4,021	0.792	0.78-0.80	0.925	$<2.2\times10^{-16}$	0.704
ROMICAT-II, n=441	0.890	0.87-0.91	0.900	$<2.2\times10^{-16}$	0.731
All test data, n=5521	0.795	0.78-0.80	0.920	$<2.2\times10^{-16}$	0.705

FHS-CT2[2]: Framingham Heart Study, participants from the second examination cycle of the Third Generation Cohort; NLST[3]: National Lung Screening Trial; PROMISE[4]: Prospective Multicenter Imaging Study for Evaluation of Chest Pain; ROMICAT-II[5]: Rule Out Myocardial Infarction using Computer Assisted Tomography II; ICC: Intraclass correlation coefficient, CI: Confidence interval. All p-values are smaller than 2.2×10^{-16}, which is the smallest positive floating-point number possible on our system.

Supplementary Table 4 Accuracy of the first and second deep learning network for localizing and segmenting the heart. Results show the median dice and the difference between the geometric center of manually and automatically segmented heart in pixel and millimeter.

	Step 1 - Heart Localization			Step 2 - Heart Segmentation		
**Cohort**	**Mean Dice**	**Median Center Difference [px]**	**Median Center Difference [mm]**	**Median Dice**	**Median Center Difference [px]**	**Median Center Difference [mm]**
FHS-CT2, n=416	0.90±0.035	6±3	9±4	0.91±0.038	6±4	9±4
NLST, n=396	0.88±0.069	11±10	15±11	0.88±0.088	9±8	13±9
Promise, n=1,045	0.87±0.064	6±4	6±3	0.91±0.050	5±5	6±4
All test data, n=1,857	0.88±0.061	7±6	9±7	0.90±0.059	6±6	8±6

FHS-CT2[2]: Framingham Heart Study, participants from the second examination cycle of the Third Generation Cohort; NLST[3]: National Lung Screening Trial; PROMISE[4]: Prospective Multicenter Imaging Study for Evaluation of Chest Pain; ROMICAT-II[5]: Rule Out Myocardial Infarction using Computer Assisted Tomography II; Results are presented as mean ± standard deviation.

Supplementary Table 5 AUC comparison for event prediction based on automatically and manually calculated calcium scores.

		AUC	SD	SE	95%CI	P-value
**NLST:** n=396; Events: ASCVD death, n=11	AI	0.69	0.08	0.08	0.54-0.84	0.0156
	Manual	0.64	0.07	0.07	0.50-0.78	0.0552
**PROMISE:** n=4,021; Events: ACM, MI, UA, n=130	AI	0.66	0.02	0.02	0.62-0.71	3.63×10^{-11}
	Manual	0.68	0.02	0.02	0.63-0.72	9.34×10^{-13}
**ROMICAT-II:** n=441; Events: ACS, n=38	AI	0.83	0.03	0.03	0.76-0.90	1.96×10^{-14}
	Manual	0.86	0.03	0.03	0.81-0.92	1.94×10^{-16}

NLST[3]: National Lung Screening Trial; PROMISE[4]: Prospective Multicenter Imaging Study for Evaluation of Chest Pain; ROMICAT-II[5]: Rule Out Myocardial Infarction using Computer Assisted Tomography II; AUC: Area under the curve, SD: Standard Deviation, SE: Standard Error, CI: Confidence interval. All p-values were calculated using a two-sided Wilcoxon Test.

1. Grundy, S. M. *et al.* 2018 AHA/ACC/AACVPR/AAPA/ABC/ACPM/ADA/AGS/APhA/ASPC/NLA/PCNA Guideline on the Management of Blood Cholesterol: Executive Summary. *Journal of the American College of Cardiology* vol. 73 3168–3209 (2019).
2. D’Agostino, R. B. *et al.* General Cardiovascular Risk Profile for Use in Primary Care. *Circulation* vol. 117 743–753 (2008).
3. National Lung Screening Trial Research Team *et al.* Reduced lung-cancer mortality with low-dose computed tomographic screening. *N. Engl. J. Med.* **365**, 395–409 (2011).
4. Douglas, P. S. *et al.* PROspective Multicenter Imaging Study for Evaluation of chest pain: rationale and design of the PROMISE trial. *Am. Heart J.* **167**, 796–803.e1 (2014).
5. Hoffmann, U. *et al.* Design of the Rule Out Myocardial Ischemia/Infarction Using Computer Assisted Tomography: a multicenter randomized comparative effectiveness trial of cardiac computed tomography versus alternative triage strategies in patients with acute chest pain in the emergency department. *Am. Heart J.* **163**, 330–8, 338.e1 (2012).
