# Supplementary material for: Deep convolutional neural networks to predict cardiovascular risk from computed tomography
Source: Nat Commun. 2021 Jan 29;12:715. doi: 10.1038/s41467-021-20966-2 (PMC7846726; doi:10.1038/s41467-021-20966-2)
Supplement: Supplementary file 2 — Reporting Summary [file 41467_2021_20966_MOESM2_ESM.pdf]

nature research

Corresponding author(s): Hugo Aerts, PhD
Phone: 617.525.7156
Email: haerts@bwh.harvard.edu

Last updated by author(s): Dec 11, 2020

Reporting Summary

Nature Research wishes to improve the reproducibility of the work that we publish. This form provides structure for consistency and transparency in reporting. For further information on Nature Research policies, see our Editorial Policies and the Editorial Policy Checklist.

Statistics

For all statistical analyses, confirm that the following items are present in the figure legend, table legend, main text, or Methods section.

n/a	Confirmed	
☐	☒	The exact sample size (*n*) for each experimental group/condition, given as a discrete number and unit of measurement
☒	☐	A statement on whether measurements were taken from distinct samples or whether the same sample was measured repeatedly
☐	☒	The statistical test(s) used AND whether they are one- or two-sided<br>*Only common tests should be described solely by name; describe more complex techniques in the Methods section.*
☒	☐	A description of all covariates tested
☐	☒	A description of any assumptions or corrections, such as tests of normality and adjustment for multiple comparisons
☐	☒	A full description of the statistical parameters including central tendency (e.g. means) or other basic estimates (e.g. regression coefficient) AND variation (e.g. standard deviation) or associated estimates of uncertainty (e.g. confidence intervals)
☐	☒	For null hypothesis testing, the test statistic (e.g. *F*, *t*, *r*) with confidence intervals, effect sizes, degrees of freedom and *P* value noted<br>*Give P values as exact values whenever suitable.*
☒	☐	For Bayesian analysis, information on the choice of priors and Markov chain Monte Carlo settings
☒	☐	For hierarchical and complex designs, identification of the appropriate level for tests and full reporting of outcomes
☒	☐	Estimates of effect sizes (e.g. Cohen's *d*, Pearson's *r*), indicating how they were calculated

Our web collection on statistics for biologists contains articles on many of the points above.

Software and code

Policy information about availability of computer code

Data collection	The data was received in the DICOM format as described in the corresponding parent multi-center trials.
Data analysis	General Software used: Python V2.7.17; pip V20.2; Nvidia GPU driver V440.100; Nvidia Cuda 10.1; Nvidia cuDNN V7.6; R V3.6.3; RStudio V1.3.1073;<br>Data processing: Python V2.7 - Packages: pydicom V1.4.2; SimpleITK V1.2.4, Numpy V1.16.6; Scipy V1.2.3; h5py V2.10.0; Results analysis: Python - Packages: scikit-learn package V0.20.4; Scipy V1.2.3; R - Packages: ICC v2.3.0; Survcomp V1.36.1; Survminer V0.4.8; Survival V3.2.3<br>Deep learning: Tensorflow-GPU V1.15.0; Keras V2.3.1;<br>Medical image analysis: FHS and NLST: 3D Slicer (V4); PROMISE and ROMICAT-II: TeraRecon workstations

For manuscripts utilizing custom algorithms or software that are central to the research but not yet described in published literature, software must be made available to editors and reviewers. We strongly encourage code deposition in a community repository (e.g. GitHub). See the Nature Research guidelines for submitting code & software for further information.

Data

Policy information about availability of data

All manuscripts must include a data availability statement. This statement should provide the following information, where applicable:

- Accession codes, unique identifiers, or web links for publicly available datasets
- A list of figures that have associated raw data
- A description of any restrictions on data availability

NLST data is available upon request from the NCI (https://biometry.nci.nih.gov/cdas/nlst/). The code of the deep learning system, as well as the trained model and statistical analysis are publicly available at our homepage https://aim.hms.harvard.edu. Although raw CT imaging data cannot be shared, all measured results to replicate the statistical analysis are shared. Furthermore, we include test samples from a publicly available data set with deep learning and expert reader heart and

calcium segmentations.

Field-specific reporting

Please select the one below that is the best fit for your research. If you are not sure, read the appropriate sections before making your selection.

[x] Life sciences [ ] Behavioural & social sciences [ ] Ecological, evolutionary & environmental sciences

For a reference copy of the document with all sections, see nature.com/documents/nr-reporting-summary-flat.pdf

Life sciences study design

All studies must disclose on these points even when the disclosure is negative.

Sample size	21,720 in total; training/tuning: 1,636; testing: 20,084
Data exclusions	FHS-CT1 training cohort: Incomplete or uninterpretable scans (n=108); Known CVD (n=150); Not interpretable by secondary post-processing (n=52); Insufficient image quality for train data set (n=693), Random remove CAC=0 images (n=857)<br>Testing cohorts:<br>FHS-CT2: Incomplete or uninterpretable scans (n=292); Known CVD (n=175); Subjects considered in train data set FHS-CT1 (n=1,759)<br>NLST: Incomplete or uninterpretable scans (n=17); Incomplete scans for deep learning (n=12); Insufficient image quality for test data set (n=10); Incomplete risk profile(n=2)<br>PROMISE: No CAC score available (n=1,839); Incomplete scans for deep learning (n=72);<br>ROMICAT-II: Incomplete or uninterpretable scans (n=11), CAC missing (n=21)<br>For details see also the Study population section in the Methods of the manuscript as well as Supplementary Figure S1.
Replication	All measured results to replicate the statistical analysis are shared. Furthermore, we include test samples from the publicly available LIDC-IDRI data set with deep learning and expert reader heart and calcium segmentations.
Randomization	The randomization of the cohorts is described in the corresponding parent multicenter trials. For NLST we had permission to include 15,000 subjects from the full cohort which we selected randomly.
Blinding	Blinding does not apply to this work as the complete available data was used in this study.

Reporting for specific materials, systems and methods

We require information from authors about some types of materials, experimental systems and methods used in many studies. Here, indicate whether each material, system or method listed is relevant to your study. If you are not sure if a list item applies to your research, read the appropriate section before selecting a response.

Materials & experimental systems

n/a	Involved in the study	
[x]	[ ]	Antibodies
[x]	[ ]	Eukaryotic cell lines
[x]	[ ]	Palaeontology and archaeology
[x]	[ ]	Animals and other organisms
[ ]	[x]	Human research participants
[x]	[ ]	Clinical data
[x]	[ ]	Dual use research of concern

Methods

n/a	Involved in the study	
[x]	[ ]	ChIP-seq
[x]	[ ]	Flow cytometry
[x]	[ ]	MRI-based neuroimaging

Human research participants

Policy information about studies involving human research participants

Population characteristics	Baseline characteristics of the participating subjects are shown in Table 1 of the manuscript. Please see also the corresponding parent multi-center trials.
Recruitment	Please see the corresponding parent multi-center trials.
Ethics oversight	Participants from all studies provided written consent. Please see the corresponding parent multi-center trials.

Note that full information on the approval of the study protocol must also be provided in the manuscript.
